# A core outcome set for lower limb orthopaedic surgery for children with cerebral palsy: An international multi‐stakeholder consensus study

**DOI:** 10.1111/dmcn.15351

**Published:** 2022-07-22

**Authors:** Hajar Almoajil, Sally Hopewell, Helen Dawes, Francine Toye, Tim Theologis

**Affiliations:** ^1^ Nuffield Department of Orthopaedics, Rheumatology and Musculoskeletal Sciences University of Oxford Oxford UK; ^2^ Department of Physical Therapy, College of Applied Medical Science Imam Abdulrahman Bin Faisal University Dammam Saudi Arabia; ^3^ College of Medicine and Health, Medicine, Nursing and Allied Health Professions University of Exeter Exeter UK; ^4^ Oxford Biomedical Research Centre Oxford UK; ^5^ Nuffield Orthopaedic Centre Oxford University Hospitals NHS Foundation Trust Oxford UK

## Abstract

**Aim:**

To develop a core set of outcome domains to be measured in clinical studies on lower limb orthopaedic surgery for ambulant children with cerebral palsy (CP) that represents the priorities of an international multi‐stakeholder group (children, parent/carers, and health professionals).

**Method:**

Potential outcome domains were identified through literature review and qualitative interviews with key stakeholders. These were scored in an international two‐round Delphi survey, using a 9‐point Likert scale. A final consensus meeting with key stakeholders agreed on the most important outcome domains and refined the core outcome set (COS).

**Results:**

One hundred and sixty‐one health professionals and 36 individuals with CP and their parents/carers rated 21 of 41 outcomes as important in the Delphi survey. The final consensus group agreed 19 outcomes within eight domains to be included in the final COS: pain and fatigue, lower limb structure, motor function, mobility (daily life activities), gait‐related outcomes, physical activity, independence, and quality of life.

**Interpretation:**

A COS for lower limb orthopaedic surgery for children with CP was developed. Incorporating this in the design of future clinical studies will provide a more holistic assessment of the impact of treatment while allowing meaningful comparisons and future synthesis of results from primary studies.

**What this paper adds:**

- Eight core outcome domains were identified as important to measure in future clinical research.
- Key stakeholders perceived pain, balance and fall, and independence as very important outcomes.
- Six contextual factors were identified as essential in surgical decision‐making.

## What this paper adds

- Eight core outcome domains were identified as important to measure in future clinical research.
- Key stakeholders perceived pain, balance and fall, and independence as very important outcomes.
- Six contextual factors were identified as essential in surgical decision‐making.

This original article is commented on by Shrader on pages 153–154 of this issue.

Plain language summary: https://onlinelibrary.wiley.com/doi/10.1111/dmcn.15557

**Abbreviations**
COMET: Core Outcome Measures in Effectiveness Trials
COS: core outcome set
ICF‐CY: International Classification of Functioning, Disability and Health: Children and Youth

Cerebral palsy (CP) is the most common cause of childhood physical disability worldwide.^1^, ^2^ Children with CP are characterized by a deterioration in gait and function caused by secondary musculoskeletal problems, which often progresses especially during the growth spurt in adolescence. Thus, orthopaedic surgery has a critical role in the management of a child with CP.^3^ Numerous reviews have reported various outcomes when evaluating the effects of lower limb surgical interventions in CP.^4^, ^5^, ^6^, ^7^, ^8^ Variations in reported outcomes challenge comparability across studies and thus translation of evidence into clinical practice, resulting in research waste.^9^, ^10^, ^11^ Standardized reporting outcome is therefore essential.

Selection of outcomes is a key component of clinical study design.^12^ Several studies on surgical interventions include outcomes of clinical interest, such as three‐dimensional gait analysis and clinical examination.^7^, ^8^ Outcomes related to safety, life impact, quality of life, and satisfaction are poorly reported in this field. It has been recommended that any reported outcomes should be relevant to all key stakeholders if they are to be used to influence clinical decisions and further research.^12^, ^13^

Given the concerns in outcome reporting, it was recognized that there is a need to develop a core outcome set (COS) for research on lower limb orthopaedic surgery in CP to improve the quality of evidence synthesis.^14^ The current drive to standardize outcome reporting in clinical research is led by the Core Outcome Measures in Effectiveness Trials (COMET) initiative.^11^ A COS is defined as an ‘agreed minimum set of outcomes that should be measured and reported in all clinical trials of a specific disease or trial publication’.^11^, ^12^

This study aimed to develop a consensus, at international level, among children, families, and health professionals on a set of core outcome domains for reporting in clinical studies on lower limb orthopaedic surgery for ambulant children with CP.

## METHOD

This study was registered in the COMET database under registration number 1236 and is retrievable on the COMET website (https://cometinitiative.org/Studies/Details/1236).^15^ A detailed study protocol defining objectives and the consensus methodology has been previously published.^14^ The study was approved by the relevant research ethics committee (19/SC/0357). The research methods are based on the COMET guideline^11^ and are reported in accordance with the Core Outcome Set – STAndards for Reporting guideline.^16^

### Scope and design

As stated in the study protocol,^14^ the scope of the proposed COS is presented in Table 1. A steering group, including healthcare professionals, researchers, adults with CP, and parents of children with CP was established to oversee the process and provide input through the different stages of the study. The two stages to identify outcomes of interest comprised (1) identification of relevant outcomes and (2) prioritization of outcome domains. Outcome constructs were derived from the World Health Organization's International Classification of Functioning, Disability and Health: Children and Youth (ICF‐CY),^17^ which has been used as an initial conceptual framework and classification tool.

**TABLE 1:** Scope of the core outcome set

Setting	Clinical and research
Health condition	Cerebral palsy
Target population	Ambulatory children and young adults (≤18 years) with cerebral palsy
Target intervention	Lower limb orthopaedic surgery

### Identification of relevant outcomes

A long‐list of relevant outcomes was identified from (1) qualitive evidence synthesis to identify outcomes from publications describing qualitative data reported by children, young adults with CP, and parents relating to lower limb orthopaedic surgical outcomes;^18^ (2) an updated scoping review to identify outcomes reported in clinical research assessing lower limb surgery was also conducted;^8^ and (3) semi‐structured interviews with 10 children and young adults, eight parents, and 10 health professionals.^19^, ^20^

**FIGURE 1:**
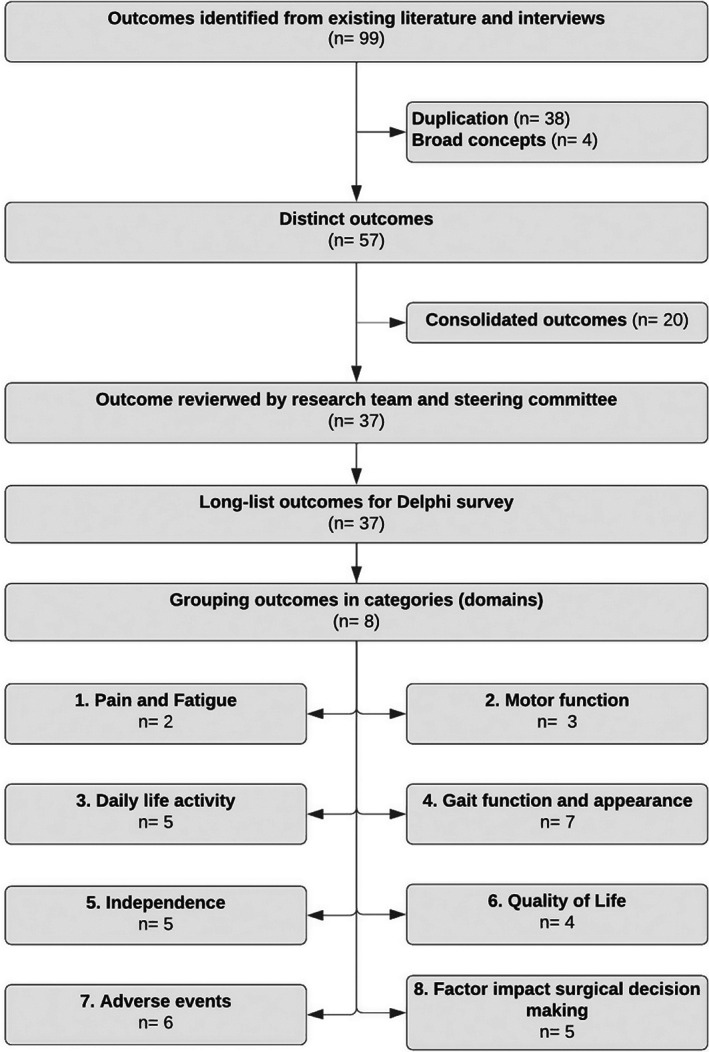
Pre‐Delphi stage of identifying long‐list outcomes.

In preparation for the outcome prioritization stage, the long‐list of outcomes was refined by the research team. The group discussed and decided whether particular outcomes duplicated each other or if they were so closely related that they could be merged into a single outcome. In addition, the research team refined the language of the outcomes to make it accessible and readable (child‐friendly) and finally allocated each outcome to a specific domain. The project steering committee then reviewed the refined list of outcome domains to ensure the readability of the outcome descriptions, and the appropriateness of the allocated domain. A total of 37 outcomes were defined, which were arranged into eight domains (Figure 1).

### Prioritization of outcome domains

**FIGURE 2:**
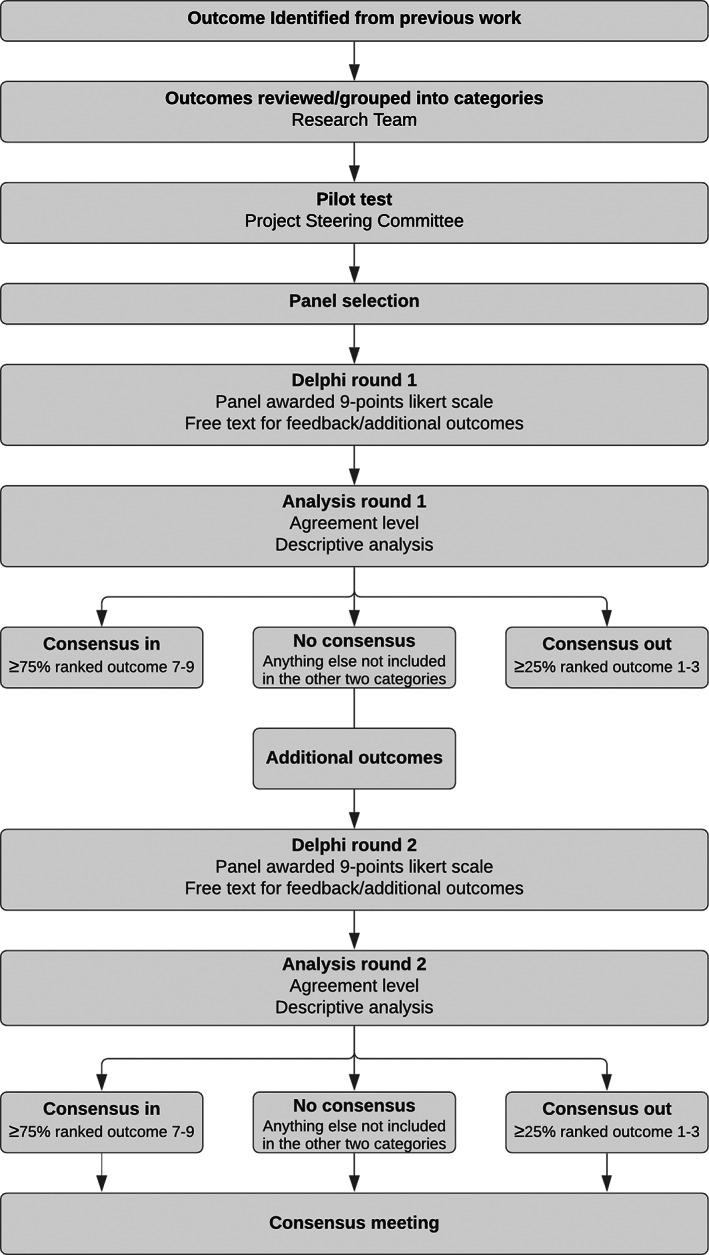
Prioritization of outcomes throughout the consensus methods.

To prioritize the 37 outcomes of lower limb orthopaedic surgery, multi‐stakeholders' international consensus methods were conducted through (1) a Delphi technique and (2) a consensus meeting (Figure 2). Detailed methods and pre‐defined consensus criteria are available in the study protocol.^14^

#### Delphi technique

A Delphi technique was conducted over two rounds, using the online Jisc platform.^21^ International participants were recruited from the two key stakeholder groups as shown in Table 2. Participants were recruited by (1) personal e‐mail invitation, (2) the snowball method, (3) relevant national and international CP charities and societies, and (4) social media (e.g. Twitter and Facebook). A child‐friendly animation was developed to explain the rationale of the study and promote children's understanding of the purpose of the COS.^22^ To take part in the survey, participants needed to have sufficient command of English to read, understand, and independently complete the questionnaires, acknowledging that children might need help from an adult. At the start of the survey, participants were asked to self‐certify that they were an ‘expert’ in at least one of the four experts' sub‐groups as presented in Table 2. Consent was inferred when the participants submitted their responses through the website.

**TABLE 2:** Delphi study stakeholders' group

Healthcare professionals	A. Healthcare practitioners A clinical qualification Currently employed by a public or private institution that provides treatment to children with cerebral palsy (CP) Have specific experience in assessing or managing lower limb musculoskeletal deformity in children with CP who receive, or have received, surgery or rehabilitation‐based interventions
	B. Clinical researchers An academic qualification Currently employed by a research organization Have current or ‘recent‐past’ experience in clinical research with studies focused on questions of clinical efficacy of lower limb orthopaedic surgery or post‐surgery‐based intervention in children with CP (i.e. authors or a co‐author on a relevant peer‐reviewed journal publication in the previous 10 years)
Children, adults, and representatives (family member/carers)	A. Children and young adults Aged <18 years, or Adults, who had surgery when they were under 18 years old Have the experience of living with CP and have had experience with at least one operation for lower limb deformity or Have the intention or are considering undergoing that treatment in the future
	B. Representative Family member (parent) or carer of a child with above criteria

In each round, participants were asked to rate the importance of each outcome for a COS using the Grading of Recommendations Assessment, Development and Evaluation (GRADE) scale of 1 to 9, where 1 represents least important and 9 represents most important.^23^ An optional open‐text box following each outcome and at the end of the round was presented for participants to add any comments or propose additional outcomes that did not appear in the list of outcomes. These additional outcomes were reviewed and categorized by the study research team, edited in appropriate plain‐language, and presented as new items in round 2.

Consensus criteria were defined a priori. Each outcome was assigned to one of three categories reflecting levels of consensus: (A) ‘consensus in’ (≥75% in one or both groups voted the outcome as ‘important for inclusion’); (B) ‘consensus out’ (≥25% voted the outcome as ‘not important’ in one or both groups); and (C) ‘no consensus’ (outcomes that did not meet either A or B criteria). Descriptive analysis in the form of percentage, median, and interquartile range of participants scoring each of outcome in each round was calculated. The data were reported separately for each stakeholder group.

#### Consensus meeting

To determine which outcomes should be recommended for inclusion in the COS for lower limb orthopaedic surgical interventions, the results of the Delphi survey were presented to 21 international panel members who participated in two online consensus meetings (7th and 14th May 2021). Panel members were (1) experienced clinicians and researchers with expertise in CP‐related research (*n* = 15), (2) adults with CP (*n* = 3), and (3) parents of children with CP (*n* = 3). Verbal consent was obtained to enable the researchers to audio‐record the meeting.

The structure of the consensus meeting included a combination of presentations of background information, discussion, and in‐depth analysis of data from the previous stage (i.e. Delphi technique), and voting. To ensure inclusion of all participants' views, small ‘break‐out’ rooms were held. Participants were allocated a priori to a break‐out room to ensure representation from the different stakeholder groups and countries. Outcomes identified as ‘consensus in’ were presented first and participants gave feedback and reflections to the whole group on their opinion about the most important outcomes and why they felt that way. This was followed by a discussion and review of outcomes deemed ‘consensus out’. Participants were asked to discuss reasons why these should not be included in the COS. The participants then discussed the ‘no consensus’ outcomes and were asked to vote anonymously on these. In particular, they were asked to distinguish whether the outcome should be (1) definitely included, (2) included as an optional outcome, or (3) definitely not included. The results were presented to the panel once the voting was completed.

## RESULTS

### Delphi technique

Given the iterative nature of the study recruitment procedure, it is not possible to calculate how many individuals received an invitation to participate during the window of recruitment. There were 111 respondents to the first‐round survey. Stakeholders were grouped into two categories. Category 1 consisted of health professionals (*n* = 93) and category 2 consisted of individuals with CP and representatives (*n* = 18). The second round was completed by 86 respondents, 68 healthcare professionals, and 18 individuals with CP and representatives. There were 66 (59.4%) participants who contributed to round 2 who had completed round 1. Table 3 outlines the demographic characteristics of the participants in Delphi rounds 1 and 2.

**TABLE 3:** Demographic characteristics of the participants in Delphi survey rounds 1 and 2

	Round 1, *n* (%)			Round 2, *n* (%)		
	Category 1 (*n* = 93)	Category 2 (*n* = 18)		Category 1 (*n* = 68)	Category 2 (*n* = 18)	
	Health professionals	Representative	Patients	Health professionals	Representative	Patients
**Current professionals**						
Physiotherapist	44 (47.3)			24 (35.2)		
Orthopaedic surgeon	34 (36.5)			32 (47)		
Researcher	9 (9.6)			10 (14.7)		
Paediatrician	2 (2.1)			1 (1.4)		
Nurse	1 (1)			—		
Paediatric physiatrist	1 (1)			—		
Human movement science	1 (1)			—		
Orthotist	1 (1)			1 (1.4)		
**Years of experience**						
>10	58 (62.3)			46 (67.6)		
5–10	20 (21.5)			16 (23.5)		
<5	15 (16.1)			6 (8.8)		
**Current place of employment**						
UK	33 (35.4)	5 (27.7)	2 (11.1)	20 (29.4)	6 (33.3)	3 (16.6)
Australia	22 (23.6)	1 (5.5)	3 (16.6)	14 (20.5)	3 (16.6)	2 (11.1)
Asia	15 (16.1)	—	—	11 (16.1)	—	—
Europe	12 (12.9)	—	—	13 (19.1)	—	—
USA	6 (6.4)	1 (5.5)	2 (11.1)	6 (8.8)	1 (5.5)	2 (11.1)
South America	3 (3.2)	—	2 (11.1)	2 (2.9)	—	—
Canada	1 (1)	—	—	1 (1.4)	—	—
Africa	1 (1)	2 (11.1)	—	1 (1.4)	1 (5.5)	—
**Sex**						
Female	61 (65.5)		4 (22.2)	38 (55.8)		2 (11.1)
Male	31 (33.3)		5 (27.7)	29 (42.6)		4 (22.2)
Prefer not to say	1 (1)			1 (1.4)		1 (5.5)
**Relationship to the child**						
Mother		7 (38.8)			8 (44.4)	
Father		2 (11.1)			3 (16.6)	
**Age (individuals with CP)**						
6–10 years		4 (22.2)	—		2 (11.1)	—
11–15 years		1 (5.5)	1 (5.5)		6 (33.3)	3 (16.6)
16–18 years		1 (5.5)	1 (5.5)		—	1 (5.5)
>18 years		3 (16.6)	7 (38.8)		3 (16.6)	3 (16.6)
Not having surgery yet		2 (11.1)	1 (5.5)		2 (11.1)	1 (5.5)
<6 months		3 (16.6)	—		1 (5.5)	—
6–12 months		1 (5.5)	1 (5.5)		—	—
1–3 years		—	—		5 (27.7)	3 (16.6)
3–5 years		1 (5.5)	2 (11.1)		1 (5.5)	1 (5.5)
>5 years		2 (11.1)	5 (27.7)		2 (11.1)	2 (11.1)
**Child's current walking ability indoors**						
Most of the time, they walk on their own		3 (16.6)	4 (22.2)		6 (33.3)	5 (27.7)
Most of the time, they walk using a crutch or walker		1 (5.5)	—		2 (11.1)	1 (5.5)
Most of the time, they walk with my support		3 (16.6)	3 (16.6)		1 (5.5)	1 (5.5)
Most of the time, they use a wheelchair		2 (11.1)	2 (11.1)		2 (11.1)	—
**Child's current walking ability outdoor**						
Most of the time, they walk on their own		1 (5.5)	3 (16.6)		3 (16.6)	2 (11.1)
Most of the time, they walk using a crutch or walker		3 (16.6)	—		2 (11.1)	2 (11.1)
Most of the time, they walk with my support		1 (5.5)	2 (11.1)		3 (16.6)	2 (11.1)
Most of the time, they use a wheelchair		4 (22.2)	4 (22.2)		3 (16.6)	1 (5.5)

Table S1 illustrates the number and percentage of participants scoring each outcome on both rounds. Appendix S1 shows the median score and interquartile range for each outcome based on the response to the first‐ and second‐round survey. Four additional outcomes were proposed during the first‐round of the Delphi survey.

On review of both rounds, there was substantial coherence between panels, with 16 outcomes achieving 75% threshold of importance and labelled as ‘consensus in’ across stakeholder groups. The main areas that reached consensus included avoiding pain, avoiding falls, maintaining static balance, and life impact (e.g. walking‐related outcomes and independence) with agreement rates ranging from 75% to 90%. Four further items related to the ‘delivery of care’, which may affect surgical decision‐making, met the threshold of importance by both stakeholder groups. These included access to physiotherapy services, communication and support from healthcare professionals, availability of information resources, and adherence to home exercises, with rates ranging from 81% to 100%.

There were differences between stakeholder groups in the outcomes that reached ‘consensus in’. Healthcare professionals rated eight outcomes that were not prioritized by the CP individuals and representatives as ‘consensus in’. For example, the use of assistive devices was rated as an important domain in both rounds (89% and 88% respectively). On the other hand, individuals with CP and representatives rated four outcomes that were not prioritized by health professionals as ‘consensus in’. For example, 89% agreed that the ability to walk with the foot flat on the ground was important and critical.

No outcome was ranked to be of limited importance (i.e. ‘consensus out’) by the health professionals; whereas four outcomes met the 25% threshold of limited importance by individuals with CP and representatives. Finally, from a total of 41 outcome domains, 11 reached ‘no consensus’ across both stakeholders’ groups. Four of these outcomes were related to adverse events and included postoperative pain syndrome, wound infection, recurrence of deformity, and time of recovery and improvement.

### Consensus meeting

From the 11 outcomes that did not reach the threshold for ‘consensus in’ in the Delphi study, three were considered important enough to be included in the core set. The results of voting for each outcome are shown in Table 4. Participants suggested that different aspects of outcomes may be important to different children. This could lead to individual outcomes not reaching the threshold for inclusion despite broad agreement from the panel that it should be included. Participants regrouped or redefined some of the outcomes. For example, fatigue captured muscle fatigue and fatigue (or tiredness) after activities.

**TABLE 4:** Voting results

Outcome	Consensus in (%)	Optional outcome (%)	Consensus out (%)
To improve the way the child looks			
To be able to stand taller or more upright	47	33	20
To have straighter and better shaped legs	87	13	—
To increase how much the child can do			
To be able to climb stairs	67	20	13
To improve participation in sports (e.g. football, swimming)	7	60	33
To improve the way the child walks after surgery			
To be able to walk faster	29	57	14
To be able to walk without using a crutch or walker	33	53	13
To improve how the child feel when interacts with other people			
The child improves friendships and relationships with other people	7	20	73
Concerns about having surgery			
Feeling pain after the surgery	20	20	60
Developing wound infection	33	40	27
Needing to have another surgery	40	33	27
Taking a long time to walk again	40	27	33

Participants discussed a wide range of outcomes that spanned the ICF‐CY framework. The group reached a high level of ‘consensus in’ for at least one outcome for each ICF‐CY component, including body function/structure, activity, and participation, such as pain, lower limb alignment, walking, and physical activity. There was lengthy discussion about outcomes related to the use of assistive devices, and participants reached a consensus that this should not be included in the COS. This was because of the insufficient evidence on decreased use of assistive devices following surgery, particularly for children classified in Gross Motor Function Classification System level III. ‘Participation’ was also discussed at length, reflecting that participants had individual areas of interest, which were not agreed upon. Thus, the group concluded that ‘physical activity’ would appropriately reflect the range of outcomes in the ‘participation’ domain.

Participants reached consensus on outcomes related to increased independence in various life roles. This reflected the high level of agreement on the Delphi study, where all outcomes related to independence reached consensus across stakeholders' groups (75–90%). However, some health professionals were uncertain whether interventions would improve those outcomes. Quality of life was thought to potentially overlap with other ICF‐CY domains, such as aspects of physical performance and social life. It was concluded that improvement in these aspects of health would probably improve the child's quality of life. However, participants favoured inclusion of self‐esteem and body image in the core set instead of the broad concept of quality of life.

There was a debate about whether adverse events should be included in the COS, noting that it was important to record adverse events in clinical research. Discussions focused on two main issues: (1) one of the main objectives of the surgery in CP is ultimately to prevent deterioration of musculoskeletal pathology and consequences; and (2) it is important to define the impact of adverse events on other outcomes (e.g. pain and function) but that adverse events do not represent an outcome per se. The final decision was that adverse event reporting would not be beneficial as a core outcome but was an essential a part of research documentation. Several reasons reflecting this decision were discussed, including (1) the need to understand both the benefit and the potential harm of surgery, (2) the need to support patients and families in decision‐making, (3) providing evidence during the development of new surgical techniques, and (4) adhering to good clinical practice guidelines.

The participants reached consensus that the list of factors (Table S1) was important in surgical decision‐making, and essential to reach optimal surgical outcomes. However, the final decision was that these were not required to be reported in all clinical research and therefore not to include them as part of the COS despite their overall importance.

At the end of the consensus meeting, 19 outcomes within eight domains were agreed for inclusion in the core outcome (domains) set for lower limb orthopaedic surgery for ambulant children with CP. These included pain and fatigue, lower limb structure, motor function, daily life activities, walking‐related outcomes, physical activity, independence, and quality of life. The final COS is represented in Table 5. A short animation was developed with the involvement of the key stakeholders to aid dissemination.^24^

**TABLE 5:** Final core outcome set for lower limb surgery for children with cerebral palsy

Core area (domain)	Outcome
Pain and fatigue	Pain
	Muscle fatigue
	Fatigue (or tiredness after activities)
Lower limb structure	Lower limb alignment
	Lower limb symmetry
Motor function	Standing position
	Balance and fall
Mobility: daily life activity	Daily life activities (e.g. self‐care, climb stairs)
Mobility: walking	Walking pattern
	Walking distance
	Maintain walking speed
	Walking endurance
	Stop using orthosis, brace, splint
Participation	Physical activity
Independence	Daily life activity
	Outdoor activity
	Walking without support from an adult
Quality of life	Self‐esteem
	Body image

## DISCUSSION

Using robust methodology in accordance with the COMET initiative recommendations,^11^ a Delphi study and a subsequent consensus meeting were used to develop an international multi‐stakeholder set of outcome domains for use in clinical research assessing lower limb orthopaedic surgery in ambulant children with CP. Implementation of this standardized reporting of core outcomes across future studies will help improve the quality of evidence synthesis.

New outcome domains relevant to different stakeholder groups were identified, which highlights the need to involve multiple stakeholders. This aligned with the findings of other COS development studies. For example, new outcomes that were of significant value to patients were identified and integrated into the core set for rheumatoid arthritis clinical trials.^25^ Jones et al.^26^ recognized that involvement of multi‐stakeholders in outcome selection enhances research relevance, value, and patient‐centredness.

The consensus meeting derived the final core set, which included 19 outcomes within eight domains. This large number of outcomes would reflect the significant challenges of selecting core outcomes in complex clinical conditions with high levels of heterogeneity, such as CP. It is important to note that different outcomes are important at different times. The clinical condition in children with CP is expected to change over time as a result of natural maturation and development.^27^ Furthermore, the evolving experience and knowledge with treating or living with CP is likely to influence stakeholders' opinions on the importance of outcome domains. Therefore, the COS is likely to evolve and develop further with time.

The COS for children with CP does not preclude the selection of other outcomes but rather represents the minimum set of outcomes recommended to be measured in future studies to ensure comparability and relevance of study outcomes. It may not be feasible for all future studies to include all of the proposed core outcomes: for example, some core outcomes such as achieving independence or improving gait require long‐term follow‐up that may not be the scope of a specific study. It is recommended that researchers should consider providing a critical account of any excluded outcomes at the study design stage.

We would recommend that adverse events of surgery should be reported in future clinical studies. Although these were not included in the COS, they affect other important outcomes, define the safety profile of the procedure, and are important in clinical decision‐making.

Although contextual factors were not prioritized during the consensus meeting, these are important in the provision of care. For example, a range of factors that are integral to shared decision‐making, such as good communication and the need for information resources, were discussed during COS development. Indeed, these factors are likely to affect patient experience and it is important to consider a holistic qualitative evaluation that goes beyond the measurement of specific outcomes. Further research in this area should enhance our understanding of factors related to provision of care in this field and their impact on outcomes.

Further research to standardize outcome measures for the core outcome domains would represent the next step in this project. Unless standard outcome measures are used, the synthesis of findings and evaluation of treatment effectiveness will continue to be a challenge^6^, ^7^, ^28^ This further research was intended and planned a priori and described in the study protocol.^14^

This is the first study to determine the COS in the field of lower limb orthopaedic surgery using a transparent international consensus process involving healthcare professionals and children with CP as key stakeholders. A further strength of this study was that the findings are underpinned by extensive evidence synthesis.

There are some limitations to consider. Guidance on multi‐language Delphi surveys has been published recently, with the aim of widening international participation in COS studies.^29^ However, the Delphi survey in this study was only conducted in English for logistical reasons. Similarly, the consensus meeting was limited to English‐speaking international experts. There is a possibility that further outcomes may have been considered if the study included non‐English speakers.

Consideration should also be given to the representativeness of the study participants. There was a relatively small number of individuals with CP or their representatives involved in the Delphi rounds. To preserve the voice of this group we separated the stakeholders' groups into two throughout the Delphi survey analysis. This is in line with other COS studies, which involved a heterogeneous panel.^30^ In addition, representation from children was less than that of adults with CP who had experienced surgery several years earlier. This may have biased the priorities of the participants with CP towards the long‐term outcomes of surgery.

Every effort was made to ensure that the outcome list for the Delphi survey was concise and that there were no duplications. A lengthy list of outcomes would have led to a lower response rate.^31^ However, the attrition rate between the two Delphi survey rounds was relatively high (40.5%).

## CONCLUSION

The proposed COS was developed for use in lower limb orthopaedic surgery for ambulant children with CP. The COS includes eight domains: pain and fatigue, lower limb structure, motor function, mobility, gait‐related outcomes, physical activity, independence, and quality of life. Implementation of the COS in future clinical studies will standardize outcome collection and reporting, which will ultimately facilitate meta‐analysis to inform clinical practice.

## Funding information

H.A. is funded for postgraduate scholarship by Imam Abdulrahman Bin Faisal University, Saudi Arabia.

## CONFLICT OF INTEREST

The authors have stated that they had no interests that might be perceived as posing a conflict or bias.

## Supporting information


**Appendix S1:** Median and IQR for each outcome within the first and second rounds Delphi survey.

**Table S1:** Descriptive analysis of outcomes included in Delphi study.

## Data Availability

Data sharing is not applicable to this article as no new data were created or analyzed in this study.

## References

[1] Eunson P. Aetiology and epidemiology of cerebral palsy. Paediatrics and Child Health 2016; 26: 367–72.

[2] Glinianaia SV , Best KE , Lingam R , Rankin J . Predicting the prevalence of cerebral palsy by severity level in children aged 3 to 15 years across England and Wales by 2020. Dev Med Child Neurol 2017; 59: 864–70. 28574167 10.1111/dmcn.13475

[3] Sharan D. Orthopedic surgery in cerebral palsy: Instructional course lecture. Indian J Orthop 2017; 51: 240–55. 28566775 10.4103/ortho.IJOrtho_197_16 PMC5439309

[4] Edwards TA , Theologis T , Wright J . Predictors affecting outcome after single‐event multilevel surgery in children with cerebral palsy: a systematic review. Dev Med Child Neurol 2018; 60: 1201–8. 30073667 10.1111/dmcn.13981

[5] Lamberts RP , Burger M , du Toit J , Langerak NG . A Systematic Review of the Effects of Single‐Event Multilevel Surgery on Gait Parameters in Children with Spastic Cerebral Palsy. PLoS One 2016; 11: e0164686. 27755599 10.1371/journal.pone.0164686 PMC5068714

[6] McGinley JL , Dobson F , Ganeshalingam R , Shore BJ , Rutz E , Graham HK . Single‐event multilevel surgery for children with cerebral palsy: a systematic review. Dev Med Child Neurol 2012; 54: 117–28. 22111994 10.1111/j.1469-8749.2011.04143.x

[7] Wilson NC , Chong J , Mackey AH , Stott NS . Reported outcomes of lower limb orthopaedic surgery in children and adolescents with cerebral palsy: a mapping review. Dev Med Child Neurol 2014; 56: 808–14. 24673603 10.1111/dmcn.12431

[8] Almoajil H , Wilson N , Theologis T , Hopewell S , Toye F , Dawes H . Outcome domains and measures after lower limb orthopaedic surgery for ambulant children with cerebral palsy: an updated scoping review. Developmental Medicine & Child Neurology 2020; 26: 1138–46. 10.1111/dmcn.14599 32567044

[9] Chalmers I , Glasziou P . Avoidable waste in the production and reporting of research evidence. The Lancet 2009; 374: 86–9. 10.1016/S0140-6736(09)60329-9 19525005

[10] Clarke M. Standardising outcomes for clinical trials and systematic reviews. Trials 2007; 8(1): 1–3. 18039365 10.1186/1745-6215-8-39 PMC2169261

[11] Williamson PR , Altman DG , Bagley H , et al. The COMET Handbook: version 1.0. Trials 2017; 18(3): 1–50. 28681707 10.1186/s13063-017-1978-4 PMC5499094

[12] Williamson P , Altman DG , Blazeby JM , et al. Developing core outcome sets for clinical trials: issues to consider. Trials 2012; 13(1): 1–8. 22867278 10.1186/1745-6215-13-132 PMC3472231

[13] Williamson P , Altman D , Blazeby J , Clarke M , Gargon E . Driving up the quality and relevance of research through the use of agreed core outcomes. J Health Serv Res Policy 2012; 17: 1–2. 10.1258/jhsrp.2011.011131 22294719

[14] Almoajil H , Dawes H , Hopewell S , Toye F , Jenkinson C , Theologis T . Development of a core outcome set for lower limb orthopaedic surgical interventions in ambulant children and young people with cerebral palsy: a study protocol. BMJ open 2020; 10: e034744‐e. 10.1136/bmjopen-2019-034744 PMC7059521 32139490

[15] Hajar Almoajil , Helen Dawes , Francine Toye , Sally Hopewell , Crispin Jenkinson , Theologis T . Development of a core outcomes set for orthopaedic interventions in ambulant children and young people with cerebral palsy. [https://cometinitiative.org/Studies/Details/1236] (accessed 30 November 2020 10.1136/bmjopen-2019-034744 PMC7059521 32139490

[16] Kirkham JJ , Gorst S , Altman DG , Blazeby JM , Clarke M , Devane D . Core Outcome Set – Standards for Reporting: the COS‐STAR Statement. PLOS Med 2016; 13(10): e1002148. 27755541 10.1371/journal.pmed.1002148 PMC5068732

[17] WHO . International classification of functioning, disability and health: children and youth version: ICF‐CY. World Health Organization. 2007: 1–322.

[18] Almoajil H , Theologis T , Dawes H , et al. Patients' and parents' views about lower limb orthopaedic surgery for ambulant children and young people with cerebral palsy: a qualitative evidence synthesis. Journal of Children's Orthopaedics 2020; 14: 562–73. 10.1302/1863-2548.14.200139 PMC7740689 33343752

[19] Almoajil H , Toye F , Dawes H , et al. Outcomes of importance to children and young adults with cerebral palsy, their parents and health professionals following lower limb orthopaedic surgery: A qualitative study to inform a Core Outcome Set. Health Expect 2022; 25(3): 925–935. 35083830 10.1111/hex.13428 PMC9122398

[20] Almoajil H , Theologis T , Dawes H , et al. Exploring the factors that influence stakeholders' expectations and subsequent perception of lower limb orthopaedic surgical outcomes for ambulant children with cerebral palsy – a qualitative study. Disability and Rehabilitation 2022; (12): 1–8. 10.1080/09638288.2021.2025272 35019783

[21] Sinha IP , Smyth RL , Williamson PR . Using the delphi technique to determine which outcomes to measure in clinical trials: recommendations for the future based on a systematic review of existing studies. PLoS Med 2011; 8(1): e1000393. 21283604 10.1371/journal.pmed.1000393 PMC3026691

[22] NDORMS . Delphi study animation. https://www.youtube.com/watch?v=0fvhHgH5V2M United Kingdom, 2019.

[23] Guyatt GH , Oxman AD , Kunz R , et al. GRADE guidelines: 2. Framing the question and deciding on important outcomes. J Clin Epidemiol 2011; 64: 395–400. 21194891 10.1016/j.jclinepi.2010.09.012

[24] NDORMS . Core outcome set in lower limb orthopaedics surgery for ambulant children with cerebral palsy. https://www.youtube.com/watch?v=ReBuT4OFy8g United Kingdom, 2022.

[25] de Wit M , Abma T , Koelewijn‐van Loon M , Collins S , Kirwan J . Involving patient research partners has a significant impact on outcomes research: a responsive evaluation of the international OMERACT conferences. BMJ open 2013; 3: e002241. 10.1136/bmjopen-2012-002241 PMC3651970 23667160

[26] Jones JE , Jones LL , Keeley TJ , Calvert MJ , Mathers J . A review of patient and carer participation and the use of qualitative research in the development of core outcome sets. PLoS One 2017; 12: e0172937. 28301485 10.1371/journal.pone.0172937 PMC5354261

[27] Vargus‐Adams J. Understanding function and other outcomes in cerebral palsy. Phys Med Rehabil Clin N Am 2009; 20: 567–75. 19643354 10.1016/j.pmr.2009.04.002 PMC2719719

[28] Amirmudin NA , Lavelle G , Theologis T , Thompson N , Ryan JM . Multilevel Surgery for Children With Cerebral Palsy: A Meta‐analysis. Pediatrics 2019; 143: e20183390. 30918016 10.1542/peds.2018-3390

[29] Alkhaffaf B , Blazeby JM , Metryka A , et al. Methods for conducting international Delphi surveys to optimise global participation in core outcome set development: a case study in gastric cancer informed by a comprehensive literature review. Trials 2021; 22: 410. 34154641 10.1186/s13063-021-05338-x PMC8218463

[30] Van ‘t Hooft J , Duffy JMN , Daly M , et al. A Core Outcome Set for Evaluation of Interventions to Prevent Preterm Birth. Obstet Gynecol 2016; 127: 49–58. 26646133 10.1097/AOG.0000000000001195 PMC7734878

[31] Gargon E , Crew R , Burnside G , Williamson PR . Higher number of items associated with significantly lower response rates in COS Delphi surveys. Journal of Clinical Epidemiology 2019; 108: 110–20. 30557677 10.1016/j.jclinepi.2018.12.010 PMC6438267

