# Supplementary material for: A core outcome set for lower limb orthopaedic surgery for children with cerebral palsy: An international multi‐stakeholder consensus study
Source: Dev Med Child Neurol. 2022 Jul 22;65(2):254–63. doi: 10.1111/dmcn.15351 (PMC10084115; doi:10.1111/dmcn.15351)
Supplement: Supplementary file 1 — Appendix S1: Median and IQR for each outcome within the first and second rounds Delphi survey. [file DMCN-65-254-s001.docx]

**Appendix S1:** Median and IQR for each outcome within the first and second rounds Delphi survey


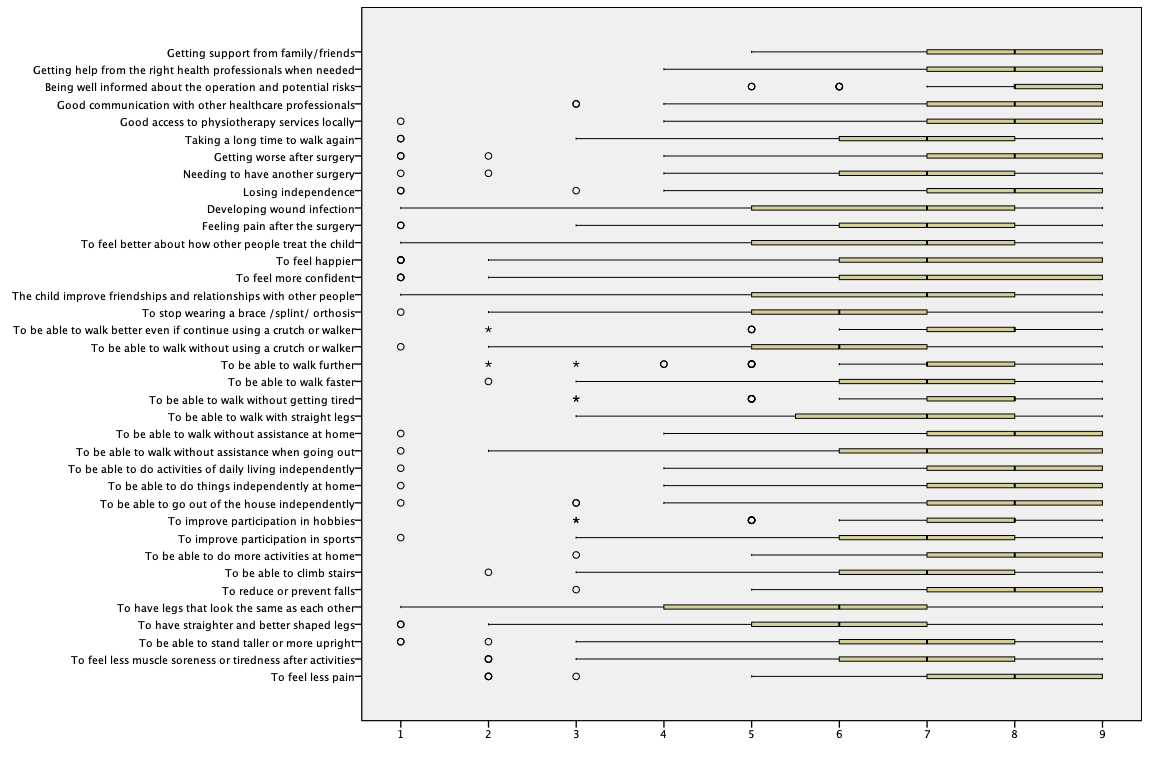


Figure 1 Round 1: Distribution of median and IQR for each outcome (health professionals)


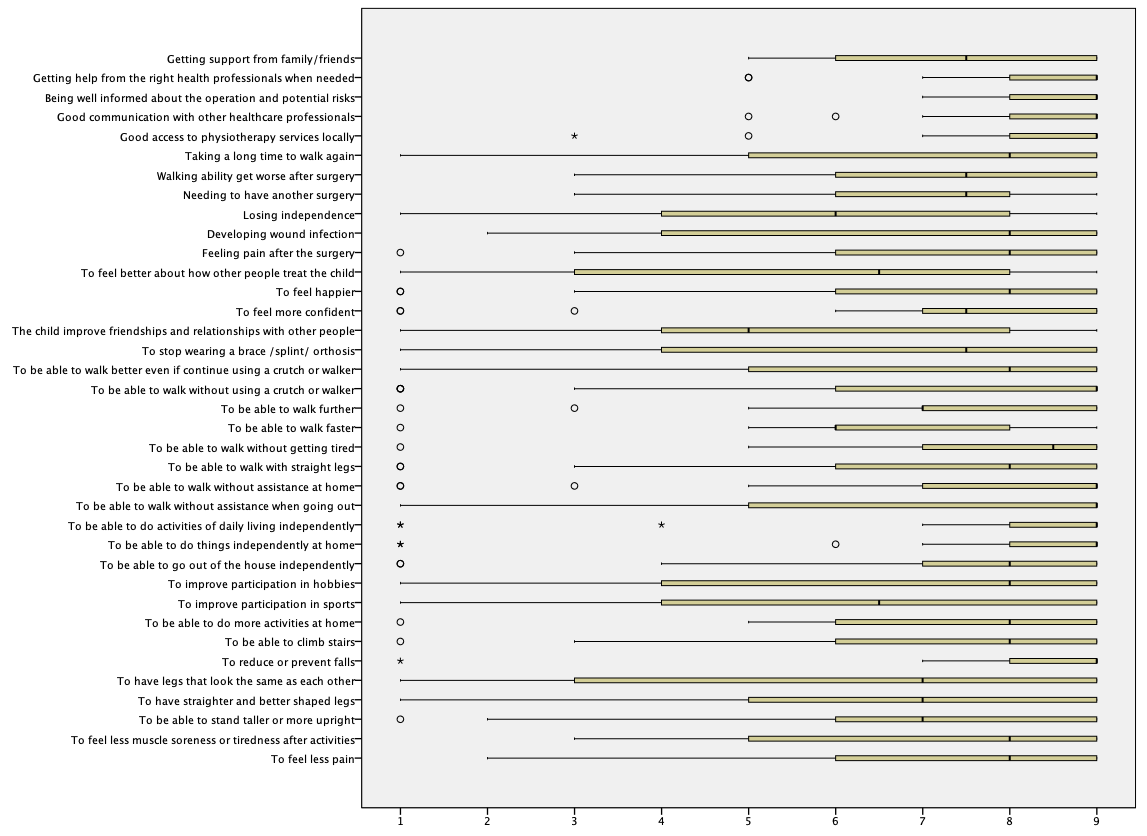


Figure 2 Round 1: Distribution of median and IQR for each outcome (individuals with CP/ representatives)


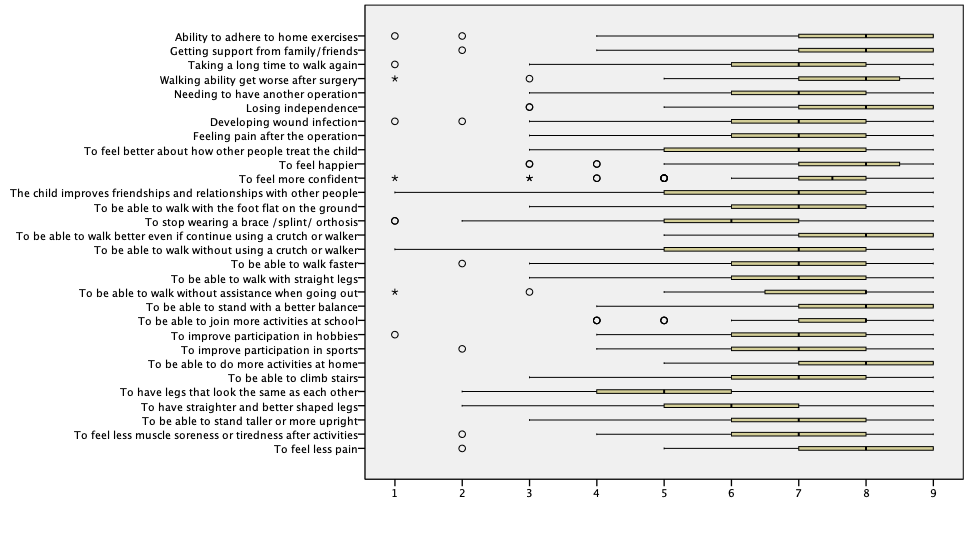
 Figure 3 Round 2: Distribution of median and IQR for each outcome (health professionals)


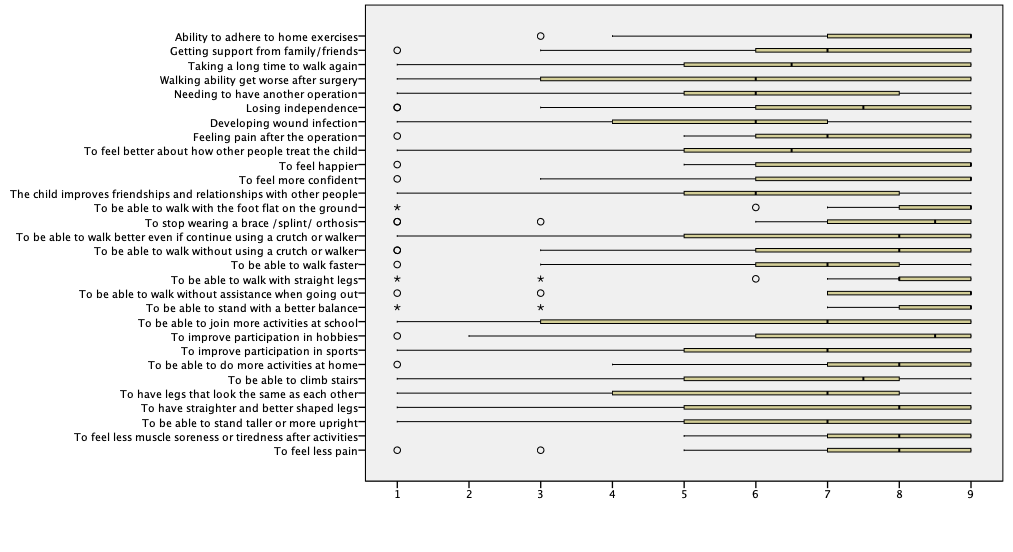


Figure 4 Round 2: Distribution of median and IQR for each outcome (individuals with CP/ representatives)
