# Supplementary material for: A core outcome set for lower limb orthopaedic surgery for children with cerebral palsy: An international multi‐stakeholder consensus study
Source: Dev Med Child Neurol. 2022 Jul 22;65(2):254–63. doi: 10.1111/dmcn.15351 (PMC10084115; doi:10.1111/dmcn.15351)
Supplement: Supplementary file 2 — Table S1: Descriptive analysis of outcomes included in Delphi study. [file DMCN-65-254-s002.docx]

TABLE S1 Descriptive analysis of outcomes included in Delphi study

| **Outcomes** | **Participant group** | **Round 1, *n* (%)** | | |  | **Round 2, *n* (%)** | | | **Category^b^** |
| --- | --- | --- | --- | --- | --- | --- | --- | --- | --- |
|  |  | **1–3** | **4–6** | **7–9** |  | **1–3** | **4–6** | **7–9** |  |
| To decrease the child’s pain and muscle soreness | | | | | | | | | |
| To feel less pain | HCP | 4 (4) | 9 (10) | 80 (86) |  | 1 (1) | 9 (13) | 58 (85) | A |
|  | Patients/rep. | 2 (12) | 3 (18) | 12 (71) |  | 2 (12) | 1 (6) | 14 (82) |  |
| To feel less muscle soreness or tiredness after activities | HCP | 6 (6) | 25 (27) | 62 (67) |  | 1 (1) | 23 (34) | 44 (65) | A* |
|  | Patients/rep. | 3 (18) | 5 (29) | 9 (53) |  | — | 3 (17) | 15 (83) |  |
| To improve the way the child looks | | | | | | | | | |
| To be able to stand taller or more upright | HCP | 7 (8) | 30 (33) | 55 (60) |  | 3 (4) | 21 (31) | 44 (65) | C |
|  | Patients/rep. | 2 (12) | 4 (24) | 11 (65) |  | 4 (24) | 2 (12) | 11 (65) |  |
| To have straighter and better shaped legs | HCP | 10 (11) | 42 (46) | 40 (43) |  | 5 (7) | 41 (60) | 22 (32) | C |
|  | Patients/rep. | 3 (17) | 4 (22) | 11 (61) |  | 2 (11) | 3 (17) | 13 (72) |  |
| To have legs that look the same as each other | HCP | 16 (17) | 43 (46) | 34 (37) |  | 13 (19) | 39 (57) | 16 (24) | B* |
|  | Patients/rep. | 5 (29) | 1 (6) | 11 (65) |  | 4 (24) | 3 (18) | 10 (59) |  |
| To increase how much the child can do | | | | | | | | | |
| To reduce or prevent falls | HCP | 1 (1) | 15 (16) | 77 (83) |  |  |  |  | A |
|  | Patients/rep. | 1 (6) | — | 17 (94) |  |  |  |  |  |
| To be able to climb stairs | HCP | 5 (5) | 34 (37) | 53 (58) |  | 1 (1) | 31 (46) | 36 (53) | C |
|  | Patients/rep. | 2 (11) | 5 (28) | 11 (61) |  | 2 (11) | 5 (28) | 11 (61) |  |
| To be able to do more activities at home | HCP | 1 (1) | 8 (9) | 84 (90) |  | — | 8 (12) | 60 (88) | A |
|  | Patients/rep. | 1 (6) | 4 (22) | 13 (72) |  | 1 (6) | 2 (11) | 15 (83) |  |
| To improve participation in sports (e.g. football, swimming) | HCP | 3 (3) | 26 (28) | 64 (69) |  | 1 (1) | 26 (38) | 41 (60) | C |
|  | Patients/rep. | 4 (22) | 5 (28) | 9 (50) |  | 3 (17) | 4 (22) | 11 (61) |  |
| To improve participation in hobbies (e.g. dancing, horse-riding) | HCP | 2 (2) | 18 (19) | 73 (78) |  | 1 (1) | 19 (28) | 48 (71) | A** |
|  | Patients/rep. | 2 (12) | 5 (29) | 10 (59) |  | 3 (17) | 3 (17) | 12 (67) |  |
| To be able to join more activities at school (e.g. PE) † | HCP |  |  |  |  | — | 12 (18) | 56 (82) | A**  B* |
|  | Patients/rep. |  |  |  |  | 5 (28) | 1 (6) | 12 (67) |  |
| To be able to stand with a better balance † | HCP |  |  |  |  | — | 9 (13) | 59 (87) | A |
|  | Patients/rep. |  |  |  |  | 2 (11) | — | 16 (89) |  |
| To increase the child’s independence | | | | | | | | | |
| To be able to go out of the house independently (without help from an adult) | HCP | 3 (3) | 8 (9) | 82 (88) |  |  |  |  | A |
|  | Patients/rep. | 2 (11) | 1 (6) | 15 (83) |  |  |  |  |  |
| To be able to do things independently at home | HCP | 1 (1) | 8 (9) | 84 (90) |  |  |  |  | A |
|  | Patients/rep. | 2 (11) | 1 (6) | 15 (83) |  |  |  |  |  |
| To be able to do activities of daily living independently (e.g. bathing, dressing and self-care) | HCP | 1 (1) | 8 (9) | 84 (90) |  |  |  |  | A |
|  | Patients/rep. | 2 (11) | 1 (6) | 15 (83) |  |  |  |  |  |
| To be able to walk without assistance when going out | HCP | 2 (2) | 22 (24) | 67 (74) |  | 2 (3) | 15 (22) | 51 (75) | A |
|  | Patients/rep. | 4 (22) | 4 (22) | 10 (56) |  | 2 (11) | 0 | 16 (89) |  |
| To be able to walk without assistance at home | HCP | 1 (1) | 15 (16) | 75 (82) |  |  |  |  | A |
|  | Patients/rep. | 3 (18) | 1 (6) | 13 (76) |  |  |  |  |  |
| To improve the way the child walks after surgery | | | | | | | | | |
| To be able to walk with straight legs | HCP | 3 (3) | 41 (45) | 48 (52) |  | 1 (1) | 24 (35) | 43 (63) | A* |
|  | Patients/rep. | 3 (17) | 4 (22) | 11 (61) |  | 2 (11) | 1 (6) | 15 (83) |  |
| To be able to walk without getting tired | HCP | 2 (2) | 14 (15) | 77 (83) |  |  |  |  | A |
|  | Patients/rep. | 1(6) | 3 (17) | 14 (78) |  |  |  |  |  |
| To be able to walk faster | HCP | 5 (5) | 35 (38) | 52 (57) |  | 3 (4) | 24 (35) | 41 (60) | C |
|  | Patients/rep. | 1 (6) | 9 (50) | 8 (44) |  | 3 (17) | 4 (22) | 11 (61) |  |
| To be able to walk further | HCP | 2 (2) | 18 (19) | 73 (78) |  |  |  |  | A |
|  | Patients/rep. | 2 (11) | 2 (11) | 14 (78) |  |  |  |  |  |
| To be able to walk without using a crutch or walker | HCP | 6 (7) | 41 (45) | 44 (48) |  | 3 (4) | 30 (45) | 34 (51) | C |
|  | Patients/rep. | 4 (22) | 1 (6) | 13 (72) |  | 3 (17) | 3 (17) | 12 (67) |  |
| To be able to walk better even if continue using a crutch or walker | HCP | 1 (1) | 9 (10) | 82 (89) |  | — | 8 (12) | 60 (88) | A** |
|  | Patients/rep. | 4 (22) | 2 (11) | 12 (67) |  | 3 (17) | 4 (22) | 11 (61) |  |
| To stop wearing a brace /splint/ orthosis | HCP | 13 (14) | 41 (44) | 39 (42) |  | 14 (21) | 28 (42) | 24 (36) | A* |
|  | Patients/rep. | 4 (22) | 4 (22) | 10 (56) |  | 3 (17) | 1 (6) | 14 (78) |  |
| To be able to walk with the foot flat on the ground † | HCP |  |  |  |  | 2 (3) | 24 (35) | 42 (62) | A* |
|  | Patients/rep. |  |  |  |  | 1 (6) | 1 (6) | 16 (89) |  |
| To improve how the child feel when interacts with other people | | | | | | | | | |
| The child improves friendships and relationships with other people | HCP | 11 (12) | 32 (34) | 50 (54) |  | 5 (7) | 27 (40) | 36 (53) | C |
|  | Patients/rep. | 4 (22) | 9 (50) | 5 (28) |  | 2 (11) | 9 (50) | 7 (39) |  |
| To feel more confident | HCP | 5 (5) | 20 (22) | 67 (73) |  | 3 (4) | 9 (13) | 56 (82) | A** |
|  | Patients/rep. | 3 (17) | 1 (6) | 14 (78) |  | 2 (11) | 4 (22) | 12 (67) |  |
| To feel happier | HCP | 6 (6) | 22 (24) | 65 (70) |  | 2 (3) | 12 (18) | 54 (79) | A** |
|  | Patients/rep. | 3 (17) | 3 (17) | 12 (67) |  | 1 (6) | 4 (22) | 13 (72) |  |
| To feel better about how other people treat the child | HCP | 11 (12) | 27 (30) | 52 (58) |  | 6 (9) | 27 (40) | 35 (51) | B* |
|  | Patients/rep. | 6 (33) | 3 (17) | 9 (50) |  | 4 (22) | 5 (28) | 9 (50) |  |
| Concerns about having surgery | | | | | | | | | |
| Feeling pain after the surgery | HCP | 6 (6) | 27 (29) | 60 (65) |  | 2 (3) | 20 (29) | 46 (68) | C |
|  | Patients/rep. | 2 (11) | 3 (17) | 13 (72) |  | 1 (6) | 5 (28) | 12 (67) |  |
| Developing wound infection | HCP | 4 (4) | 39 (42) | 50 (54) |  | 3 (4) | 26 (38) | 39 (57) | C |
|  | Patients/rep. | 3(17) | 4 (22) | 11 (61) |  | 4 (22) | 9 (50) | 5 (28) |  |
| Losing independence | HCP | 3(3) | 15 (16) | 75 (81) |  | 2 (3) | 5 (7) | 61 (90) | A** |
|  | Patients/rep. | 4 (22) | 6 (33) | 8 (44) |  | 3 (17) | 4 (22) | 11 (61) |  |
| Needing to have another surgery | HCP | 2 (2) | 35 (38) | 55 (60) |  | 5 (7) | 21 (31) | 42 (62) | C |
|  | Patients/rep. | 1 (6) | 6 (33) | 11 (61) |  | 2 (11) | 8 (44) | 8 (44) |  |
| Getting worse after surgery | HCP | 3 (3) | 11 (12) | 78 (85) |  | 2 (3) | 5 (7) | 61 (90) | A**  B* |
|  | Patients/rep. | 1 (6) | 4 (22) | 13 (72) |  | 5 (28) | 5 (28) | 8 (44) |  |
| Taking a long time to walk again | HCP | 3 (3) | 25 (27) | 65 (70) |  | 2 (3) | 21 (31) | 44 (66) | C |
|  | Patients/rep. | 2 (11) | 4 (22) | 12 (67) |  | 3 (17) | 6 (33) | 9 (50) |  |
| How important are the following factors in your decision to recommend the surgery? | | | | | | | | | |
| Good access to physiotherapy services locally | HCP | 1 (1) | 10 (11) | 82 (88) |  |  |  |  | A |
|  | Patients/rep. | 1 (6) | 1 (6) | 16 (89) |  |  |  |  |  |
| Good communication with other healthcare professionals | HCP | 2 (2) | 10 (11) | 81 (87) |  |  |  |  | A |
|  | Patients/rep. | — | 2 (11) | 16 (89) |  |  |  |  |  |
| Being well informed about the operation and potential risks | HCP | — | 6 (6) | 87 (94) |  |  |  |  | A |
|  | Patients/rep. | — | — | 18 (100) |  |  |  |  |  |
| Getting help from the right health professionals when needed | HCP | — | 6 (6) | 87 (94) |  |  |  |  | A |
|  | Patients/rep. | — | 2 (11) | 16 (89) |  |  |  |  |  |
| Getting support from family/friends | HCP | — | 8 (9) | 85 (91) |  | 1 (1) | 12 (18) | 55 (81) | A** |
|  | Patients/rep. | — | 7 (39) | 11 (61) |  | 2 (11) | 3 (17) | 13 (72) |  |
| Ability to adhere to home exercises^a^ | HCP |  |  |  |  | 2 (3) | 10 (15) | 56 (82) | A |
|  | Patients/rep. |  |  |  |  | 1 (6) | 2 (11) | 15 (83) |  |

^a^New outcomes suggested from round 1.

^b^Categories: A, ‘consensus in’ by both groups; A*, ‘consensus in’ by patients/representative (rep.; parent/carer); A**, ‘consensus in’ by healthcare professionals (HCP); B*, ‘consensus out’ by patients/representative; C, ‘no consensus’ by both groups.
